# Five-Year Forecasting Deaths Caused by Traffic Accidents in Fars Province of Iran

**DOI:** 10.29252/beat-070406

**Published:** 2019-10

**Authors:** Mahnaz Yadollahi, Saeid Gholamzadeh

**Affiliations:** 1 *Trauma Research Center, Rajaee (Emtiaz) Trauma Hospital, Shiraz University of Medical Sciences, Shiraz, Iran*; 2 *Legal Medicine Research Center, Legal medicine organization, Tehran, Iran*

**Keywords:** Accidents, Traffic, Mortality trend, Population Forecast, Health policy

## Abstract

**Objective:**

The aim of study was to conduct a time-based analysis to utilize the obtained knowledge for forecasting the trend of accidents in the future.

**Methods:**

The present study, which was conducted as a cross-sectional research, investigated deaths from traffic accidents in Fars Province during a five-year period from 2013 to 2018. The pseudo-regression model of Spline was used to predict the increase in mortality rate by 2021.

**Results:**

The forecasted values indicated a decline in deaths from traffic accidents by 2021. A total of 8020 records of accidents leading to death were included in the study. The mean mortality rate from traffic accidents in the province was approximately estimated to be 33.7 per 100,000 populations. More than half of the people who died (52.36%) were in a car, 25.57% were motorcycle riders, and 19.93% were pedestrians. The highest rate of deaths was observed in the age group of 16 to 25 years old (21.5%). The data indicated a reduction in the rate of death among car riders and pedestrians and an increase in the number of deaths among motorcycle riders. The trend of deaths occurred outside the city had been increasing while the trend of deaths occurred inside the city had been decreasing.

**Conclusion:**

The present regulations are only able to reduce a small number of deaths each year. In order to achieve a downward trend in mortality with a steeper pace, it is necessary to design and implement more intelligent standards, not merely the stricter ones.

## Introduction

Among various trauma mechanisms, traffic accidents have the highest mortality rates [1- 3]. The rate of mortality from traffic accidents is growing and its rank has risen from the tenth to the eighth cause of death in the twentieth century [4]. Every year, millions of people die, or suffer from injuries and disabilities caused by traffic accidents [5]. Traffic accidents are the fourth cause of years of life lost in developing countries such as the Middle East; in Iran the prevalence of traffic accidents is about twenty times higher than the global average, and this country is ranked as the fifth country in terms of traffic accidents [6]. Compared with the European countries, the rate of disabilities caused by traffic accidents has doubled in recent years [7].

The incidence of traffic accidents has been increasing in Iran in such a way that one person dies in roads in Iran every 19 minutes and a person is injured every two minutes [8]. The proportion of male-to-female persons injured in traffic accidents, who had been referred to a focal trauma treatment center in the south of Iran, changed from 2.7% in 2010 to 2.4% in 2014 [6]. Because of the growth of the young population and the increased use of motorcycles in urban communities, the rate of involvement of this vehicle in traffic accidents has increased to 25%, and 60% of motorcycle riders usually suffer from severe injuries in traffic accidents [8]. Fars Province, which is located in southern part of Iran, is ranked the first in terms of number of people died from traffic accidents [9].

Based on the results of a review of the literature, there is no study that have analyzed traffic accidents in Iran and predicted its trend by 2021. It is of utmost importance to prevent traffic accidents, as it helps to avoid the imposition of costs and the associated adverse effects [10]. In order to implement prevention policies, it is essential to exactly identify the factors and assess the effects of the implementation of the solutions. The aim of this study was to transform data into information and then conduct a time-based analysis to exploit the obtained knowledge for forecasting traffic accidents in the future. Through assessing the demographic characteristics and place and time of accidents, this study tried to provide effective administrative approaches for the prevention of deaths caused by traffic accidents.

## Materials and Methods

In this cross-sectional study, the sampling was performed using census method and the deaths from vehicle accidents that were registered in forensic organization over a five-year period from March 21, 2013 to March 20, 2018 were investigated. The study was carried out in Fars province with a population of 4851274 people and with a road coverage of 7367 km; the province is ranked the second in the country in terms of cost. Using a data collection form, we collected data on demographic characteristics (age, gender, and education), injuries (the major cause of death, being a driver or an occupant), and spatial and temporal characteristics of the accident (time of occurrence, location (inside or outside the city), the type of vehicle, the mechanism of the incidence of the accident, and the light status during the accident). The cases who died more than 30 days after the accident were excluded from the study due to the possible involvement of other factors in death. The institutional review board (IRB) and medical ethics approvals were obtained before study. No informed written consents were required as we used the anonymous computer based information. 

In order to determine the frequency and distribution of deaths, mean and standard deviation were calculated for quantitative variables, such as age; furthermore, relative frequency and frequency%age were used for qualitative variables. T-test and one-way-ANOVA were used for comparing the frequency of deaths by age, sex, trauma mechanism, and other factors. In addition, to predict the number of deaths by 2021, the pseudo-regression model of the spline was used to predict the number of death for 2018 to 2021. In this model, the number of deaths was calculated based on the variables of the city of death, the year of the accident and the gender of the deceased, and the shape parameter was taken into 0.1 in this model. data analyzes and the charts were plotted by R software version of 3.4.3 for windows.

## Results

The rate of mortality from traffic accidents in Fars province was estimated to be 33.7 per 100,000 people. A total of 8020 deaths from vehicle accidents were investigated. On average, 1604 people died in traffic accidents in Fars province. Foreigners accounted for 5.83% of the people who died; of all the mentioned cases, 4.71% were Afghani and 1.12% were from other countries.

Of the total number of people who died, 95.2% (7639 people) died in main roads, 2.8% (228 people) died in the suburbs of roads, such as agricultural lands and promenades, 1.15% (93 people) died in inter-provincial roads, and less than 0.01% (3 people) died in railway accidents. Considering the mechanism of the car crash, 52.36% (4200 people) who died were car riders, 25.57% (2051 people) were motorcycle riders that had the highest frequency (Table 1).

**Fig. 1:**
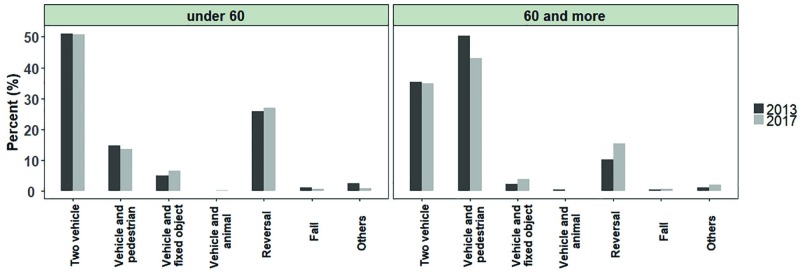
Forecasting deaths from traffic accidents by 2021

**Fig. 2:**
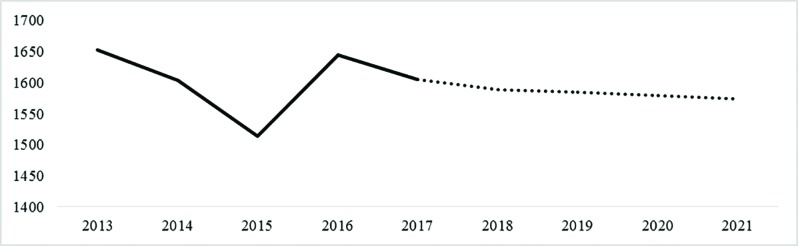
Changes in the trend of deaths from traffic accidents by age

Figure 1 shows time trend of death due to road traffic accident. The forecasted values indicated a decline in mortality from traffic accidents by 2021 (Figure 1). The highest rate of death was observed in the age group of 16 to 25 years old (21.5%) and the lowest rate was observed in the age group of 56 to 65 years old (9.73%). The results indicated no change in the age pattern of people died from traffic accidents over the past five years (Figure 2). There was a more significant balance in the distribution of age groups in vehicle occupants who died in traffic accidents.

**Fig. 3.**
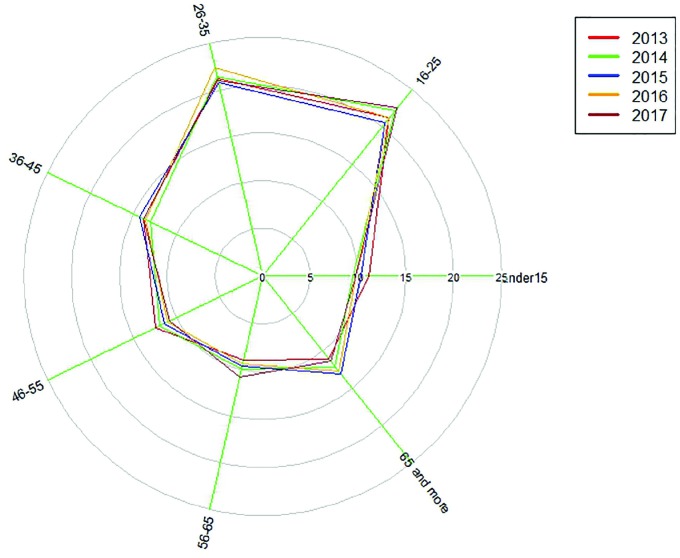
Distribution of the mechanisms of death from traffic accidents in two age groups under and over 60 years of age

Among people who died under the age of 60 years, the collision of two vehicles was the main mechanism leading to death; however, among people who died over the age of 60 years, pedestrian accidents were the main cause of death (Figure 3). Among people who died under the age of 60 years over the past five years, there has been a decrease in the frequency of deaths caused by the collision of two vehicles, the collision of a vehicle with pedestrians, and the fall of the vehicle; however, there has been an increase in the frequency of deaths caused by the collision of a vehicle with a fixed object, the collision of a vehicle with an animal, and the overturn of a vehicle. Among the people who died over the age of 60 years, there has been a decrease in the frequency of deaths caused by the collision of a vehicle with pedestrians, and the collision of a vehicle with animals; however, there has been an increase in the frequency of deaths caused by the collision between two vehicles, the collision with fixed objects, and the fall and overturn of vehicles. 

**Fig. 4:**
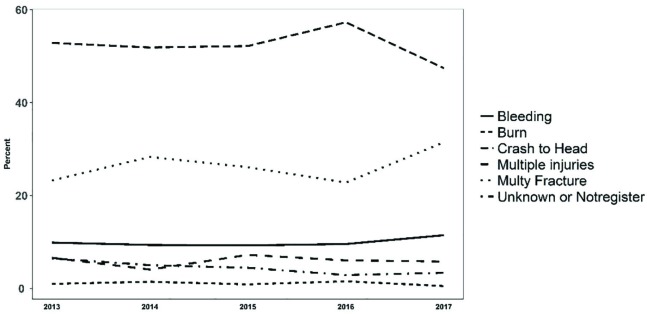
Trend of causes of death among people who died from traffic accidents

**Fig. 5:**
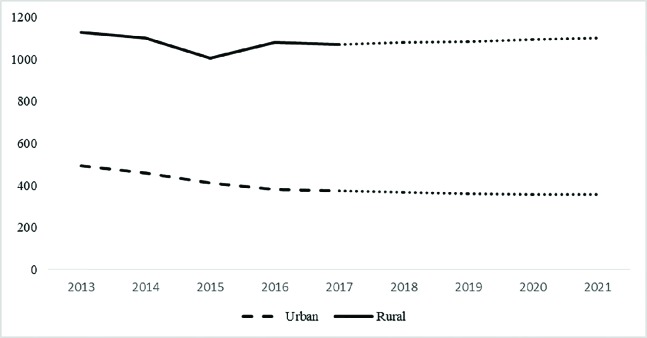
Forecasting the trend of traffic accidents leading to death inside and outside the city

**Fig. 6:**
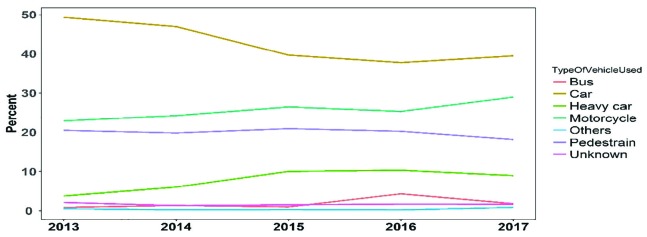
Frequency of deaths from traffic accidents by the type of vehicles used

The most common cause of death was direct damage to the head (52.36%), followed by multiple fractures (26.88%). In some cases, the cause of death was attributed to more than one factor. The analysis of the causes of death (Figure 4) indicates that during the last five years, the number of deaths caused by head injuries has been decreasing, however, multiple fractures and bleeding had increased over time. The number of deaths from traffic accidents outside the city was 67.18%. It is estimated to observe a decrease in the number of deaths inside the city and an increase in the number of deaths outside the city (Figure 5). Traffic accident outside the city is also a major cause of death in a wide range of motorcycle riders. The results indicated a reduction in deaths among people who used cars and pedestrians, and an increase in deaths among people who used motorcycles (Figure 6). 

## Discussion

Fars province is the fourth largest province in Iran in terms of size and population; in addition, the largest trauma treatment center in the south of the country is located in this province. Over the past few years, the highest rate of death from traffic accidents has been observed in this province. Based on the results of other studies, the trend of traffic accidents had been increasing from 2010 to 2016, however, the results of forecasting modeling in this study indicated a decrease in the trend of death from traffic accidents from 2016 to 2021. The role of police in monitoring and enforcement of the rules and regulations and the improvements in pre-hospital medical care were the main interventions that were effective in the reduction of mortality. As reported there is a slow decline in the frequency of mortality from accidents, which indicates that the present rules and regulations is able to reduce only a small number of deaths each year. Thus, to achieve a significant downward trend in mortality rate with a steeper slope, it is necessary to implement more effective strategies and rules, not just stricter ones.

**Table 1:** Comparison of the three main groups of people died in road accidents by demographic features and the characteristics of accidents

**Total**		**Vehicle driver **		**Motorcycle rider **		**Pedestrian**		**Value of the variable**	**Characteristic**
**Percentage**	**Frequency**	**Percentage**	**Frequency**	**Percentage**	**Frequency**	**Percentage**	**Frequency**		
20.41	1637	25.9	1088	3.85	79	28.27	452	**Female**	**Sex**
79.58	6383	74.1	3112	96.15	1972	71.71	1147	**Male**	
10.1	806	9.73	156	5.61	115	16.26	260	**Under 15 years old**	**Age group**
21.5	1725	22.51	361	35.88	736	6.88	110	**16-25 years old**	
21.3	1710	20.95	336	20.62	423	8.44	135	**26-35 years old**	
13.7	1096	13.78	221	11.41	234	10.01	160	**36-45 years old**	
11.5	924	10.85	174	9.85	202	11.76	188	**46-55 years old**	
9.73	780	10.79	173	8.9	168	13.63	218	**56-65 years old**	
12.1	968	11.41	183	8.39	172	32.9	526	**Over 65 years old**	
21.92	1758	16.33	686	18.04	370	41.71	667	**Illiterate**	**Education**
48.8	3914	46.71	1962	59.87	1228	41.34	661	**Under high school diploma**	
15.44	1238	18.14	762	15.07	309	8.88	142	**high school diploma**	
10.69	857	15.74	661	5.02	103	5.19	83	**University education**	
3.15	253	3.07	129	2	41	2.88	46	**Unknown**	
53.97	4329	54.12	2273	51.1	1048	57.47	919	**Day**	**Light status**
33.86	2716	35.12	1475	36.57	750	29.08	465	**Night**	
10.28	825	9.64	405	10.82	222	11.07	177	**Sunrise / sunset**	
1.87	150	1.12	47	1.51	31	2.38	38	**Unknown**	
52.36	4199	51.49	1763	59	1210	48.16	770	**Head injury**	**Cause of death**
10.45	838	9.64	330	10.78	221	10.19	163	**Bleeding**	
26.88	2156	27.31	935	21.31	437	28.52	456	**Multiple fractures**	
5.37	431	4.21	144	2.97	61	6.94	111	**Multiple injuries**	
1.06	85	1.93	66	0.2	4	-	-	**Burn**	
4.43	355	5.43	186	5.75	118	6.19	99	**Unknown**	
100	8020	52.36	4200	25.57	2051	19.93	1599		**Total**

The present study showed that the young age group (the legal age for obtaining a certificate is in this age range) had the highest rate of mortality; in addition, the prevalence of traffic accidents in males was four times higher than that in females. This finding is in line with the results of studies conducted in Iran and a study conducted by Singh in India that showed injuries from traffic accidents were more prevalent among young people and non-professional drivers, especially males [11-14]. One of the most important measures to reduce the incidence of deaths at this age is to provide more cohesive professional and behavioral trainings for obtaining a certificate, and raise the economic, cultural, and social status of young people. In addition, the implementation of educational programs and courses for housewives can dramatically reduce the rate of deaths from traffic accidents through influencing their husbands and children.

The present study showed that the trend of mortality from traffic accidents by age had a steady pattern over the past five years. Hence, it is possible to design and implement suitable mid-term strategic plans for risky age groups. Children and the elderly are among the people at risk. Pedestrian traffic accident is one of the main causes of the death among these age groups; thus, in order to protect them from death it is necessary to facilitate transportation through the construction of pedestrian underpasses, and electric bridges and elevators instead of stepping bridges [15].

The present study showed that most of the accidents resulting in the death in Fars province occurred during the first hours of the night, which could be attributed to people’s stresses at the end of the day, the haste and hurriedness to do daily work, reduced vision during these hours, as well as special cultural features of citizens and the geographical conditions of Fars province. This finding is inconsistent with the results of a study by Caraccans *et al*. in Turkey that showed an increase in deaths from traffic accidents from 4 to 8 am [16]. Therefore, the occurrence of more traffic accidents in specific hours does not necessarily indicate a higher rate of mortality. The present study showed that the majority of road traffic accidents leading to death occurred in the first half of the year, especially in the summer. This finding is in line with the results of studies in Iran [17] and other studies in the world [18] that measured the impact of seasons on accidents.

The results of this study showed that the collision of cars with each other and pedestrian accidents were the main mechanisms of death in Fars province. This finding is consistent with another study that reported the mentioned mechanisms as the main causes of death in trauma patients who referred to the focal center for trauma treatment in the south of Iran in Fars province [19].

Head injury is still the leading cause of death in traffic accidents, however, there is also an increase in death from bleeding. Bleeding can be due to the increase in the incidence of injuries in the abdomen and multiple fractures in the pelvic region. In a study on people injured in traffic accidents in 2013, the injuries in the chest and abdomen had a high frequency among people died in traffic accidents in south of Iran [20]. It seems that controlling bleeding and trying to retrieve the lost blood, because of their non-invasive nature, is by far the best way to prevent the death of people injured in road traffic accidents. In order to significantly decrease mortality among motorcycle riders, it is recommended to promote the use of motorcycle chambers and implement facilitating regulations, such as the allocation of a special route to motorcycles. The increase in the use of public transport facilities will help to reduce the utilization of cars and other similar vehicles and consequently reduce accidents for both users and pedestrians.

The deaths of foreigners impose huge financial burden on the health system annually. The deaths from traffic accidents may reduce to some extent through preventing human trafficking in the country's transportation system [21]. The observed increase in deaths due to the collision of a vehicle with a fixed object and car overturning indicates a reduction in people carefulness during driving over the last few years. Relying heavily on vehicle capabilities and the emotions provoked during a high-speed driving after the use of alcohol or drugs can be a major contributor to the incidence of road traffic accidents. In spite of the definite decline in the death rate in pedestrian accidents, since the large number of people injured in such accidents is under the age of 15 and over the age of 65, we must admit that pedestrians play a very significant role in pedestrian accidents. As the first step in designing a mid-term plans to prevent traffic accidents, it is essential to change the age pattern of mortality that has been fixed over the past five years through providing specialized trainings.

The number of traffic accidents has declined in urban areas and has increased in roads between the cities, but the collision between two vehicles in the urban areas can largely result in death. Ranked next to pedestrian’s crashes, car crashes are the second major cause of death in the cities. Providing privileges for drivers who drive at a safe speed at urban areas can be effective in reducing such accidents. In addition, the intra-urban mortality can be decreased via designing interurban bus route in such a way that the designed routes have the minimum cross sections with the routes intended for other vehicles and pedestrians [15].

This study had several strengths; for example, it precisely estimated the number of people died in traffic accidents, it had a large sample size, and its results are suitable for making health policies. In this study, injured patients who had been hospitalized for up to 30 days after the accident and who died afterwards were excluded from the study and this may increase the probability of the selection bias. Moreover, due to the lack of information about the number of Fars native people died due to accidents in other provinces, it was not possible to accurately estimate the mortality rate. Given the geographical location of Shiraz and the existence of subsidiary passages that cover areas in the south of the country, it is necessary to implement interventions to increase the safety of roads leading to the center of the province. Among the likely interventions, we may not the followings: dividing long roads into smaller parts via utilizing constructing road tolls, using telecommunication electronic systems to calculate the average speed of a vehicle per time, rebuilding the covering layer of roads, and installing warning signs in beltways. The other prospective goal of the researchers is to conduct research projects such as the present study and discover the life pattern of people who died through the use of new methods for machine learning to perform other preventive measures.

In conclusion, the present regulations are only able to reduce a small number of deaths each year. In additional, it is estimated to observe a decrease in the number of deaths occurred in urban accidents and an increase in the number of deaths suburban. In order to achieve a downward trend in mortality with a steeper pace, it is necessary to design and implement more intelligent standards.

## References

[1] Curtis KA, Mitchell RJ, Chong SS, Balogh ZJ, Reed DJ, Clark PT (2012). Injury trends and mortality in adult patients with major trauma in New South Wales. Med J Aust.

[2] Harrison-Felix CL, Whiteneck GG, Jha A, DeVivo MJ, Hammond FM, Hart DM (2009). Mortality over four decades after traumatic brain injury rehabilitation: a retrospective cohort study. Arch Phys Med Rehabil.

[3] MacKenzie EJ (2000). Epidemiology of injuries: current trends and future challenges. Epidemiol Rev.

[4] Lozano R, Naghavi M, Foreman K, Lim S, Shibuya K, Aboyans V (2012). Global and regional mortality from 235 causes of death for 20 age groups in 1990 and 2010: a systematic analysis for the Global Burden of Disease Study 2010. Lancet.

[5] Beadenkopf WG, Polan AK, Boek WE, Korns RF, James G (1956). An epidemiological approach to traffic accidents. Public Health Reports.

[6] Yadollahi M, Ghiassee A, Anvar M, Ghaem H, Farahmand M (2017). Analysis of Shahid Rajaee hospital administrative data on injuries resulting from car accidents in Shiraz, Iran: 2011–2014 data. Chinese journal of traumatology.

[7] Rasouli MR, Nouri M, Zarei MR, Saadat S, Rahimi-Movaghar V (2008). Comparison of road traffic fatalities and injuries in Iran with other countries. Chin J Traumatol.

[8] Unicef (2014). Road traffic injuries in Iran and their prevention, a worrying picture.

[9] Haghparast-Bidgoli H, Saadat S, Bogg L, Yarmohammadian MH, Hasselberg M (2013). Factors affecting hospital length of stay and hospital charges associated with road traffic-related injuries in Iran. BMC Health Serv Res..

[10] Ghaem H, Hajipour M, Tabatabaee HR, Yadollahi M, Izanloo F (2017). Time Series Analysis of Mortalities Resulting from Car Accidents in the Injured Individuals Hospitalized in Shiraz Shahid Rajaee Hospital During 2010-2016. Trauma Monthly.

[11] Fallahzadeh H, Dehgani A (2011). Epidemiology of road traffic mortality and injuries in Yazd, Iran during 2003-2008. Chin J Traumatol.

[12] Sheikhghomi S, Rahimi-Movaghar V, Jafarpour S, Saadat S (2015). Epidemiology and short-term mortality in traumatic patients admitted to Shariati Hospital in Iran between 2012 and 2013. Chin J Traumatol.

[13] Singh D, Singh SP, Kumaran M, Goel S (2016). Epidemiology of road traffic accident deaths in children in Chandigarh zone of North West India. Egyptian journal of forensic sciences.

[14] Moafian G, Aghabeigi MR, Heydari ST, Hoseinzadeh A, Lankarani KB, Sarikhani Y (2013). An epidemiologic survey of road traffic accidents in Iran: analysis of driver-related factors. Chin J Traumatol.

[15] Marzaleh MA, Naseri M, Naseri K (2015). Evaluation of factors affecting pedestrians’ safety margin on the streets without traffic signs. Safety Promotion and Injury Prevention.

[16] Karacasu M, Er A, Bilgiç S, Barut HB (2011). Variations in traffic accidents on seasonal, monthly, daily and hourly basis: Eskisehir case. Procedia-Social and Behavioral Sciences..

[17] Peymani P, Heydari ST, Hoseinzadeh A, Sarikhani Y, Hedjazi A, Zarenezhad M (2012). Epidemiological characteristics of fatal pedestrian accidents in Fars Province of Iran: a community-based survey. Chin J Traumatol.

[18] Nofal FH, Saeed AA (1997). Seasonal variation and weather effects on road traffic accidents in Riyadh city. Public Health.

[19] Abbasi H, Bolandparvaz S, Yadollahi M, Anvar M, Farahgol Z (2017). Time distribution of injury-related in-hospital mortality in a trauma referral center in South of Iran (2010-2015). Medicine (Baltimore).

[20] Yadollahi M, Anvar M, Ghaem H, Bolandparvaz S, Paydar S, Izianloo F (2017). Logistic regression modeling for evaluation of factors affecting trauma outcome in a level I trauma center in Shiraz. Iranian Red Crescent Medical Journal.

[21] Yadollahi M, Jamali B (2019). Severity and injury characteristics among matched hospitalized motorcycle drivers and their passengers. Chin J Traumatol.

